# Effects of TCMC on Transformation of Good Health Status to Suboptimal Health Status: A Nested Case-Control Study

**DOI:** 10.1155/2015/259727

**Published:** 2015-08-06

**Authors:** Tian Wang, Jieyu Chen, Xiaomin Sun, Lei Xiang, Lin Zhou, Fei Li, Changsong Lin, Pingping Jiang, Shengwei Wu, Ya Xiao, Jingru Cheng, Ren Luo, Yanyan Liu, Xiaoshan Zhao

**Affiliations:** ^1^Department of Traditional Chinese Medicine, Nanfang Hospital, Southern Medical University, Guangzhou, Guangdong 510515, China; ^2^School of Traditional Chinese Medicine, Southern Medical University, Guangzhou, Guangdong 510515, China; ^3^Endocrinology Department, Nanfang Hospital, Southern Medical University, Guangzhou 510515, China; ^4^Department of Rheumatic Diseases, The First Affiliated Hospital, Guangzhou University of Chinese Medicine, Guangzhou 510405, China

## Abstract

To explore the effects of traditional Chinese medicine constitution (TCMC) on transformation of good health status to suboptimal health status (SHS), we conducted a nested case-control study among college students in China. During the 18-month mean follow-up time, 543 cases of SHS (42.7%) occurred in 1273 healthy students. There was a significant (*P* = 0.000) and marked reduction in SHMS V1.0 total score in the case group at the 18-month follow-up (69.32 ± 5.45) compared with baseline (78.60 ± 4.70), but there was no significant change in the control group. Conditional logistic regression analysis showed that respondents reporting Yin-deficiency and Qi-deficiency were, respectively, 2.247 and 2.198 times more likely to develop SHS, while tendency to Yin-deficiency and tendency to Damp-heat were, respectively, 1.642 and 1.506 times more likely to develop SHS. However, the Balanced Constitution was a significant protective factor (OR 0.649; *P* < 0.05). Altogether, these findings demonstrate that Yin-deficiency, Qi-deficiency, tendency to Yin-deficiency, and tendency to Damp-heat appeared to induce a change in health status to SHS, while the Balanced Constitution seemed to restrain this change. We conclude that regulating the unbalanced TCMC (such as Yin-deficiency and Qi-deficiency) may prevent a healthy status developing into SHS or lead to the regression of SHS.

## 1. Background

The traditional Chinese medicine constitution (TCMC) is defined as the state of a population or an individual, with relative stability in function, structure, and metabolism, formed during growth, development, and aging under the influence of environmental factors and genetic background [1]. In 2009, criteria for the classification and criterion of TCMC were published by the State Administration of Traditional Chinese Medicine. According to these criteria, the TCMC is divided into nine types, which can be divide into Balanced Constitution and eight unbalanced constitutions, including Qi-deficiency, Qi-stagnation, Yang-deficiency, Yin-deficiency, Blood-stasis, Damp-heat, Phlegm-dampness, and Inherited Special Constitution (Table 1) [1]. Previous studies have presented evidence in the associations between TCMC and disease, like metabolic syndrome [2], anxiety/depression [3, 4], coronary heart disease [5], carotid artery plaque [6], and primary dysmenorrhea [7]. However, there is no direct evidence that TCMC are more likely to predispose an individual to a change in health status.

Suboptimal health status (SHS) is the intermediate state between health and disease, which is characterized by perception of health problems, general weakness, and low energy within a period of 3 months and is regarded as a subclinical, reversible stage of chronic disease [8]. The prevention and intervention strategies for SHS are similar to those of preventive, predictive, and personalized medicine (PPPM), which is targeted toward improving health, preventing disease, and treating early-stage disease [9]. Over the years, the concept of SHS has become widely accepted in many countries other than China, such as Japan [10], Australia [11], and Papua New Guinea [12]. Our previous investigation had showed that SHS was applicable to 46.0% of our total survey population [13]. Multiple population-based studies have shown increases in the occurrence of SHS [14, 15]. SHS now represents a growing health challenge worldwide. Still, the etiology of subhealth and the mechanism of its development are still unclear.

Previously, others have reported that in the cross-sectional study unbalanced constitutions are more susceptible to SHS [16]. Accordingly and in view of the importance of TCMC and health status, we put forward the hypothesis that unbalanced constitution might promote a transformation of good health status to SHS. Thus, we conducted a nested case-control study among college students in China, which aimed to examine the existence of associations between TCMC and SHS.

## 2. Methods

### 2.1. Study Design and Population

The study was approved by the ethics committee of Nanfang Hospital in Guangzhou, China (2012), LunShenZi (number 035), which also approved the consent procedure. All participants provided informed consent.

**Figure 1:**
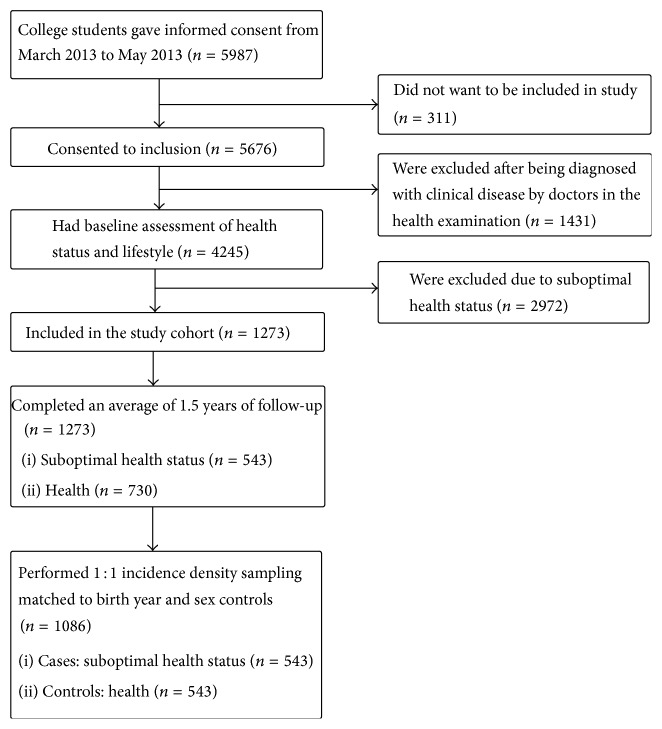
Flow of eligible participants in study.

The subjects in this nested case-control study were identified from an existing clinic-based cohort established at the Nanfang Hospital in Guangzhou and were consecutively enrolled from the cohort of college students who signed the informed consent form March 2013 to May 2013. Of these, 311 students had to move elsewhere and thus dropped out of the study. In total, 5676 students (1973 men and 3703 women) attended a health examination in the hospital, which included medical history-taking, physical examination, blood hematology and biochemistry analyses, resting electrocardiography, abdominal ultrasonography, and chest radiography. The diagnosis of disease was based on clinical criteria. Any participants diagnosed with clinical disease during the health examination (1431 students) were excluded. The medical report and the Suboptimal Health Measurement Scale Version 1.0 (SHMS V1.0) were used to evaluate health status, and any participants with SHS (2972 students) were also excluded. The detailed process is shown in Figure 1.

### 2.2. Study Cohort

After the health condition evaluation, 1273 healthy students (477 men and 796 women, average age 19.09 ± 1.08) were enrolled in our cohort and followed up for 18 months.

### 2.3. Health Assessments

#### 2.3.1. Baseline

The questionnaire was a combination of self-designed questionnaire items and standardized questionnaires. The self-designed questionnaire items parts are the general demographic characteristics (including age, gender, BMI, smoking, and drinking). The standardized questionnaires parts are composed of “Suboptimal Health Measurement Scale Version 1.0 (SHMS V1.0)” and “Constitution in Chinese Medicine Questionnaire (CCMQ)” to assess the participants' TCMC. The uniform instructions used in the test were given by the trained investigators in the scene investigation. The assessment was completed by each volunteer within 30 min. Then the questionnaire was checked by our research staff in order to make sure the data were complete and to remove any nonqualifying questionnaires immediately.

#### 2.3.2. SHS Assessment

The second part was evaluation of SHS, which was performed according to the clinical guidelines for SHS published by the China Association of Chinese Medicine (CACM) [8]. To measure SHS, we used the SHMS V1.0, which is a multidimensional, self-report symptom inventory developed by our research group in China [17]. The SHMS V1.0 consists of 39 items in total, 35 of which are divided between 3 symptom dimensions (physiological, psychological, and social) and 10 factors. The total scores were then calculated. A low total score represents a high likelihood of SHS (i.e., poor health). Prior to surveying, participants had attended an annual unit health examination in hospital, comprising medical history, a physical examination, blood haematology and biochemical analyses, rest ECG, and chest radiography. After exclusion of participants diagnosed with clinical disease in the health examination by clinical doctors, threshold values for SHS within the physiological, psychological, and social dimensions of SHMS V1.0 were 68, 67, and 67, respectively. If participants were not in SHS with respect to any of these three dimensions (physiological, psychological, and social), they were considered healthy [13, 18, 19].

#### 2.3.3. CCMQ Assessment

The third part was the Constitution in Chinese Medicine Questionnaire (CCMQ), which was used to evaluate each volunteer's TCMC. The CCMQ is regarded as the standard measurement of TCMC types recommended by CACM and has been shown in previous studies to have good validity and reliability [20]. The CCMQ consists of 60 items in total, with each item having 5 answer categories, based on the frequency of each symptom (none, occasionally, sometimes, constantly, and always). In the data analysis, these categories were assigned to a five-point scale (none: 1, occasionally: 2, sometimes: 3, constantly: 4, and always: 5). The 60 items were divided into 9 constitution scales. If the score for every unbalanced constitution subtype was ≥40, the person was considered to have an unbalanced constitution; if the score for every unbalanced constitution subtype was ≥30 to <40, they were considered to have a tendency to an unbalanced constitution, and if the score was <30, they were considered not to have an unbalanced constitution. Finally, if the score for every unbalanced constitution subtype was <30 and that of the Gentleness subtype was ≥60, the person was considered to have the Balanced Constitution [21].

### 2.4. Identification of Cases and Controls

After the 18-month follow-up, the same evaluation methods of health status (the physical examination and the questionnaires) were repeated. Participants were asked if they had experienced any uncomfortable symptoms during the previous month.

The remaining students who still had good health status (judged for baseline) were considered for the control group. In total, 730 students were considered to have good health status, and from these, we randomly selected 543 controls frequency-matched by their sex, age, and grade.

### 2.5. Data Analysis

All the data were analyzed by SPSS (version 13.0; SPSS Inc., Chicago, IL, USA). Descriptive analyses were performed on all variables and on the prevalence of SHS and CCMQ. Pearson *χ* ^2^ test was used to compare the independent variables versus the dependent variables. Owing to the category matching of the case-control study design, we performed conditional logistic regression analysis to estimate the odds ratios and 95% confidence intervals for the risk of SHS outcome associated with TCMC. All *p* values were two sided and we considered values <0.05 to be statistically significant.

## 3. Results

### 3.1. General Characteristics of Subjects

The detailed distribution of the patients' demographic characteristics is summarized in Table 2. In total, 543 cases and 543 controls were included. No significant difference was observed between cases and controls for sex, age, BMI, smoking status, or alcohol intake (*p* = 1.000, *p* = 0.200, *p* = 0.209, *p* = 1.000, and *p* = 0.247, resp.), indicating that there was no significant difference in the additional variables of smoking status, BMI, and alcohol intake between the case and control groups that might have confounded results.

### 3.2. Distribution of TCMC in Cases and Controls

The numbers and corresponding percentage distribution of TCMC types in the groups are presented in Table 3. There were significant differences in TCMC distributions between cases and controls among Balanced Constitution (*χ* ^2^ = 10.022, *p* = 0.002), Qi-deficiency (*χ* ^2^ = 7.990, *p* = 0.018), and Yin-deficiency (*χ* ^2^ = 12.570, *p* = 0.002). The percentages of Qi-deficiency and Yin-deficiency (Yes or Tendency) were lower in the control group than in the case group, for example, the Qi-deficiency were taking account of 5.2% in case, while 2.6% in controls; and the Yin-deficiency 10.1% in cases while 5.3% in controls. However, the controls (58.7%) accounted for a higher percentage than the cases (49.2%) in Balanced Constitution.

### 3.3. Mean Scores for the Individual Dimensions of SHMS V1.0 at Baseline and the End of Follow-Up

During the 18-month mean follow-up time, 543 cases of SHS (42.7%) occurred in 1273 healthy students. The scores for the individual dimensions of SHMS V 1.0 at baseline and the end of follow-up are shown in Table 4. There was a marked reduction in SHMS V 1.0 total score in the cases at 18-month follow-up (69.32 ± 5.45) than at baseline (78.60 ± 4.70) and this difference was significant (*t* = 33.17, *p* = 0.000). There was also a significant marked reduction from baseline versus end of follow-up for three of the individual dimensions: physiological (74.97 ± 8.25 versus 80.63 ± 6.51; *t* = 15.68, *p* = 0.000), psychological (64.43 ± 7.92 versus 76.15 ± 6.06; *t* = 30.90, *p* = 0.000), and social (67.05 ± 10.01 versus 78.70 ± 6.91; *t* = 25.08, *p* = 0.000). However, there was no significant change in the control group.

### 3.4. Conditional Logistic Regression Analysis to Explore the Association between TCMC and SHS

Conditional logistic regression analysis was used to estimate the odds ratios and 95% confidence intervals for the risk of SHS outcome associated with the TCMC, and the results are shown in Table 5. After additional adjustments for sex, age, BMI, smoking status, and alcohol intake, respondents reporting Yin-deficiency and those reporting Qi-deficiency were, respectively, 2.247 and 2.198 times more likely to develop SHS, while those with tendency to Yin-deficiency and those with tendency to Damp-heat were, respectively, 1.642 and 1.506 times more likely to develop SHS. However, the Balanced Constitution was a significant protective factor (OR = 0.649; *p* < 0.05).

## 4. Discussion

To our knowledge, this is the first study investigating the association between TCMC and transformation of health status. In this study, we found that there was an apparent decline in the SHMS V1.0 total score in the case group with 18-month follow-up (78.60 ± 4.70) compared with baseline (69.32 ± 5.45), and this difference was significant (*t* = 33.17, *p* = 0.000). There was also a significant reduction from baseline to the end of follow-up in the three dimensions of SHMS V1.0, including physiological, psychological, and social. However, there was no significant difference in the control group. There were also significant differences in Balanced Constitution, Yin-deficiency, and Qi-deficiency between the two groups as measured at baseline. Conditional logistic regression analysis showed that Yin-deficiency and Qi-deficiency appeared to promote transformation of health status to SHS, while Balanced Constitution seemed to be a protective factor against SHS.

There are two forms of TCMC [22]. One is considered the physiological constitution, also known as the Balanced Constitution. According to TCMC theory, Balanced Constitution is a constitution with equilibrium between Yin and Yang. People with the Balanced Constitution are energetic and well-proportioned and find it easy to adapt to their natural environment and social conditions [23]. One study showed that, compared with people with an unbalanced constitution, people with the Balanced Constitution have better physical function [24]. This is a possible explanation as to why Balanced Constitution was the dominant constitution in the control group in this study and was a protective factor against SHS.

The other TCMC form is considered a pathological or unbalanced constitution. Qi-deficiency is one of the most common unbalanced constitutions and is characterized by fatigue, lowly and weak voice, dizziness, and tendency to colds. According to TCM theory, Qi is the basic material that constitutes the human body and maintains the activities of life. Qi provides the energy for the whole body, constitutes blood, and is responsible for metabolic functioning of the body, functioning of all the body organs. All the physiological functions and pathological changes in the human body, including various psychological changes, are considered to be the result of Qi motion [25]. TCM believes that “overwork damages Qi”; thus, with rapid social development, people's lifestyles have altered, with these changes being characterized by intense competition, stress, and fewer breaks, all of which can be summarized as “overwork,” and these factors may result in SHS [26]. A previous study showed that Qi-deficiency is caused by imbalances of substance exchanges between blood and interstitial fluid, leading to an increase in the interstitial liquid volume or a decrease in nutrients and retention of metabolic wastes in interstitial fluid [27]. Compared with Balanced Constitution, there are 11 specific different metabolites of Qi-deficient constitution: betaine, proline, leucine, glutamine, serine, histidine, alanine, nicotinamide nucleotide, isoleucine, aspartic acid, and inositol [28]. These may be the material basis of SHS.

With the social economy development and high pace of life, there are great changes of lifestyle having been taken among people, especially in the youth, such as a tendency to having poorer habits (burning the midnight oil, little exercise, etc.). These habits can lead to complex changes within the body and may eventually lead to damage of the Yin of the body. In the TCM theory, Yin-deficiency means the shortage of body fluid and the decline of moisture, which could result in the hyperactivity of yang, with body organs' function hyperfunction. Yin-deficiency is associated with SHS with varied clinical manifestations, like tinnitus, amnesia, insomnia, dreaminess, dry mouth, and so forth. Many studies have shown that compared with the Balanced Constitution, people with Yin-deficiency have endocrine disorder [29] contributing to a significant rise of serum cortisol and adrenocorticotropic hormone. Subsequent research [30] further suggested that people with Yin-deficiency have downregulation of several genes closely connected with energy metabolism. As the main characteristic of SHS is fatigue, which is the consequence of lack of energy, this may explain why individuals with Yin-deficiency have a higher risk of transformation to SHS.

Improper diet mainly impairs the spleen and stomach, leading to dysfunction of them. And spleen dysfunction is the foundation of dampness: the root of the problem of Damp-heat is spleen deficiency. And the main function of spleen is producing nutrient Qi. Spleen deficiency will result in deficiency of Qi and blood. And tendency to Damp-heat will develop to Damp-heat. So tendency to Damp-heat might be associated with change from good health status to SHS.

A previous study showed that the unbalanced TCMC (such as Yang-deficiency, Yin-deficiency, Qi-deficiency, Qi-stagnation, Blood-stasis, and Damp-heat) may be risk factors for SHS by cross-sectional study [31]. In our study, only Qi-deficiency, Yin-deficiency, tendency to Yin-deficiency, and tendency to Damp-heat made a large contribution to the transformation of good health status to SHS, which is not in accordance with previous findings. This may be due to several reasons. First, we found that the proportions of unbalanced TCMC in the case group were significantly higher than in the control group, but conditional logistic regression analysis showed that only those with Qi-deficiency, Yin-deficiency, tendency to Yin-deficiency, and tendency to Damp-heat had a higher risk of developing SHS. This indicates that they maybe have a far greater impact on changes of health status than the other unbalanced TCMCs. Second, the follow-up time may not have been long enough. The change in health status was a gradual process. We found that there were significant differences in TCMC distributions between cases and controls, and the percentage of unbalanced TCMC in the case group was higher than that of the control group. It is possible that we will obtain more meaningful results after increasing the length of follow-up time in our future studies.

## 5. Conclusion

To our knowledge, this is the first study to find that Qi-deficiency, Yin-deficiency, tendency to Yin-deficiency, and tendency to Damp-heat are significantly associated with the risk of SHS incidence, and they may be able to promote the transformation from good health status to SHS, while the Balanced Constitution may be a protective factor, using a nested case-control study. Therefore, we conclude that adjusting unbalanced TCMC (such as Yin-deficiency and Qi-deficiency) may prevent good health status changing to SHS or lead to the regression of SHS.

## Strengths and Limitation of This Study

- According to TCMC theory, specific constitution is correlated to certain disease. However, there has been no prospective study to investigate the possible causal relationship between TCMC and health status change.
- To our knowledge, this is the first report exploring the nature relationship between TCMC and health status change, using a nested case-control study.
- Qi-deficiency, Yin-deficiency, tendency to Yin-deficiency, and tendency to Damp-heat are significantly associated with the risk of SHS incidence, and they may be able to promote the transformation from good health status to SHS or lead to the regression of SHS.

## Figures and Tables

**Table 1:** The characteristics of the TCM constitution.

TCM constitution	Body configuration	External appearance	Personality traits
Balanced Constitution	Moderate body shape	Ruddy complexion, good sleep, energetic, regular pulse, light red tongue with thin coating	Sunny

Qi-deficiency	Muscle grows flabby	Low voice, shortness of breath, lassitude, easy to sweat, weak pulse, reddish tongue with tooth print	Introvert

Yin-deficiency	Tall and lean	Flush face, preferring cool drink, thready and weak pulse, feverish sensation in the palms and soles, reddish tongue with thin or no coating	Irritable

Yang-deficiency	Usually fat	Pale face, cold hands and feet, fear of cold, deep and weak pulse, preferring hot food and drink, fat and tender tongue with tooth print and white coating	Introvert

Phlegm-dampness	Usually fat	Light yellow complexion, more oil on the face, slippery pulse, more sweat and phlegm, greasy and fat tongue with white coating	Patient and mild

Damp-heat	Moderate or thin	More oil on the face, somnolence, bitter taste in mouth, feel somatic heavy, thirst, short and yellow urine, viscous or dry defecate, red tongue with yellow and greasy coating, slippery and rapid pulse	Irritable

Blood-stasis	Fat or thin	Hyperpigmentation, dark and dim skin, easy to have a ecchymosis, dim lip and tongue, uneven pulse	Irritable, forgetful

Qi-stagnation	Usually thin	Depression, emotional fragility, being worried, light red tongue with thin coating, stringy pulse	Introvert, sensitive, Worrying

Inherited Special Constitution	Congenital anomalies	Urticaria, pharyngeal itching, nasal obstruction, sneeze, asthma	Allergic, handicapped

**Table 2:** Baseline characteristics of cases and controls of study participants.

	Cases (*n* = 543)	Controls (*n* = 543)	Level of significance
Sex			
Men	188 (34.6%)	188 (34.6%)	0^†^ *p* = 1.000
Women	355 (65.4%)	355 (65.4%)	
Mean (SD) age, years	18.97 (1.07)	18.89 (1.06)	−1.28^*∗*^ *p* = 0.200
BMI			
Baseline	19.99 (2.71)	20.21 (3.24)	1.26^*∗*^ *p* = 0.209
Smoker			
No	539 (99.3%)	539 (99.3%)	0^†^ *p* = 1.000
Yes	4 (0.7%)	4 (0.7%)	
Alcohol intake			
Never	279 (51.4%)	254 (46.8%)	4.14^†^ *p* = 0.247
Little	220 (40.5%)	240 (44.2%)	
Sometimes	44 (8.1%)	47 (8.7%)	
Often	0 (0)	2 (0.4%)	
Always	0 (0)	0 (0)	

^*∗*^ *t*-test for continuous variables; ^†^ *χ* ^2^ test for dichotomous variables.

**Table 3:** Distribution of TCMC at baseline in case and control groups.

TCMC	Classification	Cases	Controls	*χ* ^2^	*p *
Balanced Constitution	Yes	267 (49.2)	319 (58.7)	10.022	0.002
	No	276 (50.8)	224 (41.3)		

Qi-stagnation	Yes	4 (0.7)	6 (1.1)	0.785	0.675
	Tendency	23 (4.2)	19 (3.5)		
	No	516 (95.0)	518 (95.4)		

Qi-deficiency	Yes	28 (5.2)	14 (2.6)	7.990	0.018
	Tendency	72 (13.3)	55 (10.1)		
	No	443 (81.6)	474 (87.3)		

Yang-deficiency	Yes	47 (8.7)	35 (6.4)	1.951	0.377
	Tendency	37 (6.8)	36 (6.6)		
	No	459 (84.5)	472 (86.9)		

Yin-deficiency	Yes	55 (10.1)	29 (5.3)	12.570	0.002
	Tendency	68 (12.5)	51 (9.4)		
	No	420 (77.3)	463 (85.3)		

Blood-stasis	Yes	8 (1.5)	7 (1.3)	0.230	0.891
	Tendency	32 (5.9)	29 (5.3)		
	No	503 (92.6)	507 (93.4)		

Phlegm-dampness	Yes	21 (3.9)	11 (2.0)	3.262	0.196
	Tendency	38 (7.0)	37 (6.8)		
	No	484 (89.1)	495 (91.2)		

Damp-heat	Yes	42 (7.7)	39 (7.2)	3.603	0.165
	Tendency	63 (11.6)	45 (8.3)		
	No	438 (80.7)	459 (84.5)		

Inherited Special Constitution	Yes	14 (2.6)	13 (2.4)	0.146	0.930
	Tendency	21 (3.9)	19 (3.5)		
	No	508 (93.6)	511 (94.1)		

TCMC: traditional Chinese medicine constitution.

**Table 4:** The mean scores for the individual dimensions of SHMS V 1.0 at baseline and the end of follow-up.

	Baseline	18-month follow-up	Difference	Paired *t*-test	*p* value
Cases					
SHMS total score (0–100)	78.60 (4.70)	69.32 (5.45)	9.28 (6.52)	33.17	0.000
Physiological dimension	80.63 (6.51)	74.97 (8.25)	5.65 (8.41)	15.68	0.000
Psychological dimension	76.15 (6.06)	64.43 (7.92)	11.72 (8.84)	30.90	0.000
Social dimension	78.70 (6.91)	67.05 (10.01)	11.65 (10.83)	25.08	0.000
Controls					
SHMS total score (0–100)	80.80 (5.40)	80.97 (5.40)	−0.17 (6.10)	−0.67	0.506
Physiological dimension	82.87 (6.80)	83.32 (6.60)	−0.45 (0.32)	−1.392	0.165
Psychological dimension	78.60 (6.83)	78.60 (7.00)	0 (8.09)	0.01	0.996
Social dimension	80.50 (7.19)	80.48 (7.40)	0.02 (8.30)	0.05	0.960

**Table 5:** Conditional logistic regression analysis to explore the association between TCM and SHS.

Exposed factors	Presence	Cases	Controls	Unadjusted		Adjusted	
				OR (95% CI)	*p*	OR (95%)^*∗*^	*p*
Balanced Constitution	Yes	132	286	0.669 (0.522–0.856)	0.001	0.649 (0.505–0.834)	0.001

Yin-deficiency	Yes	107	48	2.161 (1.340–3.486)	0.002	2.247 (1.386–3.642)	0.001
	Tendency	100	69	1.555 (1.038–2.329)	0.032	1.642 (1.091–2.473)	0.017

Qi-deficiency	Yes	73	20	2.195 (1.128–4.272)	0.021	2.198 (1.126–4.290)	0.021
	Tendency	91	40	1.422 (0.973–2.078)	0.069	1.415 (0.966–2.073)	0.075

Blood-stasis	Yes	50	17	1.43 (0.414–3.152)	0.796	1.202 (0.431–3.352)	0.725
	Tendency	59	28	1.120 (0.653–1.921)	0.680	1.152 (0.669–1.985)	0.610

Phlegm-dampness	Yes	72	32	1.915 (0.923–3.974)	0.081	2.063 (0.980–4.344)	0.057
	Tendency	88	39	1.048 (0.653–1.683)	0.845	1.113 (0.688–1.800)	0.664

Qi-stagnation	Yes	43	4	0.667 (0.188–2.362)	0.667	0.716 (0.201–2.553)	0.607
	Tendency	84	15	1.211 (0.659–2.223)	0.538	1.232 (0.668–2.273)	0.504

Damp-heat	Yes	126	45	1.120 (0.718–1.745)	0.618	1.139 (0.728–1.783)	0.569
	Tendency	73	51	1.441 (0.970–2.142)	0.070	1.506 (1.010–2.247)	0.045

Inherited Special Constitution	Yes	28	13	1.018 (0.508–2.301)	0.839	1.144 (0.536–2.441)	0.728
	Tendency	41	26	1.113 (0.589–2.105)	0.741	1.142 (0.601–2.169)	0.685

Yang-deficiency	Yes	71	48	1.368 (0.872–2.146)	0.172	1.360 (0.864–2.140)	0.185
	Tendency	77	51	1.051 (0.659–1.677)	0.833	1.049 (0.656–1.677)	0.843

^*∗*^Adjusted for sex, age, BMI, smoking status, and alcohol intake.
